# Anti-Inflammatory Activity of 3, 5-Diprenyl-4-hydroxyacetophenone Isolated from *Ageratina pazcuarensis*

**DOI:** 10.3390/ijms232315012

**Published:** 2022-11-30

**Authors:** Sarai Rojas-Jiménez, María Salud Pérez-Gutiérrez, Ernesto Sánchez-Mendoza, Rubria Marlen Martínez-Casares, Nimsi Campos-Xolalpa, María Guadalupe Valladares-Cisneros, David Osvaldo Salinas-Sánchez

**Affiliations:** 1 Doctorado en Ciencias Biológicas y de la Salud, Universidad Autónoma Metropolitana-Xochimilco, Calzada del Hueso 1100, Coyoacán 04960, Ciudad de México, Mexico; 2 Departamento de Sistemas Biológicos, Universidad Autónoma Metropolitana-Xochimilco, Calzada del Hueso 1100, Coyoacán 04960, Ciudad de México, Mexico; 3 Facultad de Ciencias Químicas e Ingeniería, Universidad Autónoma del Estado de Morelos, Avenida Universidad 1001, Cuernavaca 62209, Morelos, Mexico; 4 Centro de Investigación de Biodiversidad y Conservación, Universidad Autónoma del Estado de Morelos, Avenida Universidad 1001, Cuernavaca 62209, Morelos, Mexico

**Keywords:** anti-inflammatory activity, 3, 5-diprenyl-4-hydroxyacetophenone, *Ageratina pazcuarensis*, 12-O-tetradecanoylphorbol 13-acetate, cytokines, J774A.1 macrophages, DPPH assay

## Abstract

Inflammation is implicated in a wide variety of physiological and pathological processes. Plants are an important source of active anti-inflammatory compounds. The compound 3, 5-diprenyl-4-hydroxyacetophenone (DHAP) was isolated from the dichloromethane extract of the aerial parts of *Ageratina pazcuarensis* by chromatography and identified by spectroscopic (IR, NMR) and spectrometric (GC-MS) methods. Anti-inflammatory activity was evaluated on ear edema mouse induced with 12-O-tetradecanoylphorbol 13-acetate (TPA) at 2 mg/ear. The antioxidant activity of DHAP was determined using DPPH assay. Cell viability was tested in J774A.1 macrophages, the levels of NO, TNF-α, IL-1β, IL-6, and IL-10 production in macrophages stimulated with lipopolysaccharide (LPS), and membrane lysis induced by hypotonic solution in erythrocytes were evaluated. DHAP diminished the ear edema mouse in 70.10%, and it had scavenger effect against the radical with IC_50_ of 26.00 ± 0.37 µg/mL. Likewise, 91.78 µM of this compound inhibited the production of NO (38.96%), IL-1β (55.56%), IL-6 (51.62%), and TNF-α (59.14%) in macrophages and increased the levels of IL-10 (61.20%). Finally, 25 and 50 µg/mL DHAP provided the greatest protection against erythrocyte membrane lysis. These results demonstrate that DHAP has anti-inflammatory activity.

## 1. Introduction

Inflammation is a natural bodily response to injury, being a protective mechanism that aims to eliminate the cause of damage and tissue reparation. This response leads to the restoration of homeostasis, even though inflammation is a physiological process that produces discomfort for the patient such as pain, fever, headaches, and muscle stiffness [1]. Nevertheless, if the inflammatory process persists chronic inflammation might result, and this is associated with different disorders such as allergies, diabetes, obesity, cancer, and cardiovascular diseases [2,3].

Various drugs are used to control and suppress excessive inflammation. They also reduce pain and fever, however, they have adverse effects, such as nausea or stomach ulcers, that produce internal bleeding and anemia, severe vomiting, and allergic reactions, among others. For this reason, the search for new compounds with high anti-inflammatory activity and fewer side effects than drugs used in the clinic is very important.

Plants used in traditional medicine are a source of phytochemical compounds with anti-inflammatory activity. Since ancient times, plants have played an important role in the healthcare system [4] and have been used as primary agents in the treatment of illness. In many countries plants are still used for the treatment of different diseases. *Ageratina* is a genus belonging to the Astereaceae family that includes 248 species, most of them being perennial herbaceous plants and a few of them being shrubs. Several species of *Ageratina* have been used in traditional medicine to treat various health problems, such as infections, arthritis, pain, allergies, parasites, and stomach ache, and have also been used as antivirals and immunostimulants [5,6]. *A. pichinchensis* is used to treat mycoses [7], *A. petiolare* to treat stomach aches, and *A. macrophyllum* is used against general muscular aches [8]. The metabolites present in *Ageratina* genus including flavonoids, benzyl benzoates, benzofurans, chromenes, and terpenoids. The biological studies of extracts and/or compounds isolated from these species possess several biological activities, including antioxidant, antimicrobial, and anti-inflammatory [9].

One species of *Ageratina genus*, *Ageratina pazcuarensis* (Kunth) R. M. King & H. Rob is used in traditional medicine to treat pain and in women after childbirth. This vegetal specie is commonly known as axihuitl is a shrub that grows in pine-oak forest is an endemic Mexican herb. This plant is found in the north zone of Morelos state near to the Tepozteco National Park [8].

In this study, we isolated 3, 5-diprenyl-4-hydroxyacetophenone (DHAP) from the dichloromethane extract of *A. pazcuarensis*. DHAP was first isolated from *Senecio gallicus* [10], and in 2013, it was obtained from *A. pichinchensis,* and its antinociceptive and gastroprotective activities were evaluated [11,12].

The aim of this study was to evaluate the activities of DHAP using in vivo and in vitro assays. This work reported for first time that DHAP is founded in *A. pazcuarensis.*

## 2. Results

### 2.1. Structure of DHAP

**Figure 1:**
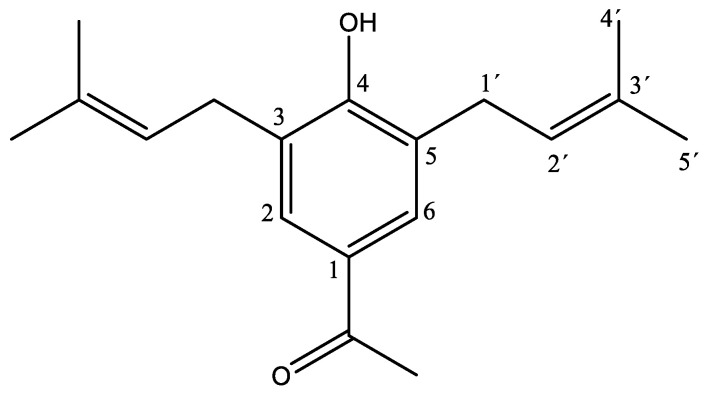
DHAP chemical structure.

White crystals (m.p. 88.5–89.6 °C) were isolated from a dichloromethane extract of *A. pazcuarensis* on the fraction of 85:15 of hexane:ethyl acetate (yield 0.036%). Spectroscopic and spectrometric analysis indicated that the compound corresponded to 3, 5-diprenyl-4-hydroxyacetophenone (Figure 1), as previously reported [10,11]. The spectroscopic and spectrometric data were as follows: ^1^H NMR (600 MHz, CDCl_3_) δ 7.63 (H2, H6), 5.94 (OH), 5.31 (H2′), 3.37 (H1′), 2.53 (CH_3_-CO), 1.78 (H4′, H5′). ^13^C NMR (151 MHz, CDCl_3_) δ 197.34 (CO), 157.41 (C4), 135.16 (C3′), 129.95 (C1), 128.83 (C2, C6), 127.07 (C3, C5), 121.36 (C2′), 29.66 (C1′), 26.33 (CH_3_CO), 25.82 (C5′), 17.94 (C4′). FT-IR, 3382.41 cm^−1^ (OH), 1746.93 cm^−1^ (C=O), 1643.93 cm^−1^ (C=C), 2965.23 cm^−1^ (=C-H), 2883.43 and 2920.24 cm^−1^ (C-H), and 1370, 1661 and 1642 cm^−1^ (C=C), 1083 cm^−1^ (C-O), CG, Rt = 7.8 min and 272.18 m/z. λ_max_ = 227.43 nm and 282.37 nm (the spectra NMR, IR, and MS are included in Supplementary Material, Figures S1–S8). 

### 2.2. Acute Anti-Inflammatory Activity: Edema Induced by TPA

Dichloromethane extract of the aerial parts of *A. pazcuarensis* was obtained, it was evaluated on ear edema in mice induced with TPA, at a concentration of 2 mg/ear. This extract diminished the edema by 77.75 ± 6.50%, the effect being close to that obtained with indomethacin (IND) (86.89 ± 5.2%).

Table 1 shows the effect of DHAP on ear edema induced with TPA in mice at doses of DHAP of 0.5, 1.0 and 2.0 mg/ear, the effect of this compound is dose dependent. At 2 mg/ear, the activity of DHAP was similar to IND.

### 2.3. The Antioxidant Activity

The antioxidant activity of DHAP was measured by DPPH assay. This compound had scavenger effect against the radical with IC_50_ of 26.00 ± 0.37 µg/mL, and ascorbic acid (positive control) presented IC_50_ of 60.81 ± 1.33 µg/mL. These results showed that the antioxidant activity of DHAP is higher than the ascorbic acid.

### 2.4. Levels of Nitric Oxide (NO) and Cytokines

The viability of macrophages treated with DHAP at seven different concentrations from 3.67 to 734.24 μM was evaluated on J774A.1 macrophages. The cytotoxic activity of this compound showed the IC_50_ value was 436. 2 µM; thus, in the other experiments a concentration of 91.78 μM was used, at which DHAP displayed 9% of cytotoxicity.

**Figure 2:**
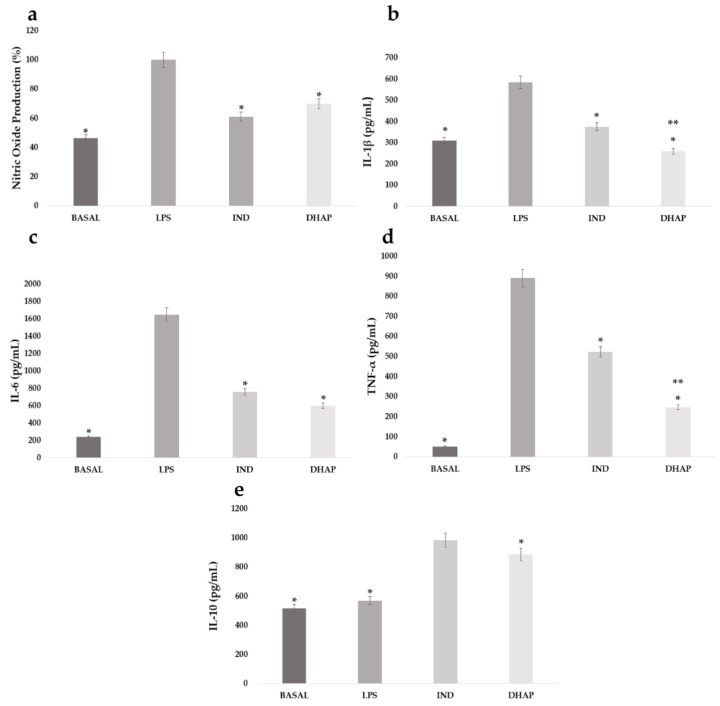
Effect of DHAP and IND at concentrations of 91.78 µM (25 μg/mL) and 4.79 µM (17.1 μg/mL), respectively in J774A.1 macrophages stimulated with LPS on the production of NO (**a**), IL-1β (**b**), IL-6 (**c**), TNF-α (**d**) and IL-10 (**e**). The graph represents the mean ± standard error of three independent experiments (each experiment with *n* = 4) * *p* < 0.01 and ** *p* < 0.05 statistically significant difference compared (**a**–**d**) with LPS, and IL-10 (**e**), DHAP group was compared with the IND group.

Macrophages stimulated with LPS were treated with DHAP or IND at concentrations of 91.78 μM and 47.79 μM, respectively. The results showed that DHAP diminished the production of NO by 38.96% (Figure 2a), which was similar to the reduction observed with IND (30.22%).

The effect of DHAP on the production of the pro-inflammatory cytokines IL-1β, IL-6, and TNF-α, and the anti-inflammatory cytokine IL-10, was determined in LPS-stimulated macrophages treated with DHAP or IND. These two compounds decreased the levels of IL-1β by 55.56% and 35.8%, respectively (Figure 2b), and IL-6 production was inhibited by 51.62% and 43.83%, respectively (Figure 2c). The concentration of TNF-α also decreased by 59.14% and 33.75%, respectively (Figure 2d). Additionally, it was found that DHAP induced an increase in the release of anti-inflammatory interleukin, IL-10 (61.20%). This increase was lower than that obtained with IND (Figure 2e).

### 2.5. Membrane Stabilization Property

The protective effect of DHAP on the membrane lysis in human red blood cells [13] was determined at concentrations of 25 to 400 µg/mL (Table 2), and the results showed that at 25 to 100 μg/mL, the effect of DHAP is independent of the concentration, the protection diminished at the highest concentrations (200 and 400 μg/mL).

## 3. Discussion

White crystals were isolated from the dichloromethane extract of *A. pazcuarensis* and identified as DHAP. Their anti-inflammatory activity was demonstrated in models in vivo and in vitro. The organism produces free radicals and they conduce to oxidative stress, which is related to different diseases such as cancer, diabetes mellitus, inflammation, etc. Antioxidant compounds could be capturing free radicals to reduce the damage produced by these chemical species [14,15,16]. The present study shows that DHAP can scavenge some free radicals. Therefore, these results suggest that the anti-inflammatory activity might be due to there being to antioxidant capacity of this compound [17]. TPA is a phorbol ester that can be used in a single dose to induce ear edema, involving erythema, edema and leucocytes, and neutrophils infiltration.

It activates protein kinase C [18], T lymphocytes, phospholipase A_2_, and the production of pro-inflammatory cytokines such as IL-1β, IL-6 and TNF-α [19].

We found that the topical administration of DHAP inhibited the skin inflammation induced by TPA, which indicates that this compound might inhibit the production of pro-inflammatory mediators.

NO has a short half-life and plays several physiological and biochemical functions in the body. This compound is produced from arginine and oxygen by four isoforms of nitric oxide synthases (NOS): neuronal NOS, endothelial NOS (eNOS), inducible NOS (iNOS), and expression of eNOS. NO has both inflammatory and anti-inflammatory effects; thus, NO promotes and regulates the pro-inflammatory cytokines COX-2 and KB nuclear factor (NFκB) [20], and it induces the formation of reactive nitrogen species involved in tissue damage. A good strategy in the treatment of inflammatory diseases is the use of NO inhibitors [21], and we observed that DHAP inhibited NO production in macrophages stimulated with LPS.

Interleukin 1β is a pro-inflammatory cytokine. It plays an important role in the host-defense response to injury and infection [22] and is released by several cell types, including macrophages and monocytes.

Another important cytokine that is produced in response to infection and tissue damage is IL-6. This interleukin is released by multiple cell types, including macrophages and T and B cells [23], and plays an important role in the inflammatory process. At certain levels of concentration, IL-6 controls the acute inflammatory response, but in chronic inflammation it is a pro-inflammatory cytokine [24].

TNF-α is a cytokine produced by macrophages and lymphocytes [25] which was initially identified to cause the necrosis of tumors. However, it has since been found that this cytokine plays a role as a pathological component in autoimmune diseases, promoting the production of different inflammatory molecules such as cytokines and chemokines [26].

IL-10 is produced by many activated immune cells, such as macrophages, neutrophils, and dendritic cells, among others. The main function of IL-10 is as an anti-inflammatory, restricting the damage caused by the inflammatory response [27] inhibiting the expression of inflammatory cytokines [28].

Research has led to the development of new drugs to block the production of NO, IL-1β, IL-6, and TNF-α, and increase the levels of IL-10, which are targets in the prevention and treatment of inflammatory diseases. In this study we found that DHAP inhibited the levels of NO, IL-1β, IL-6, and TNF-α, and enhanced the production of IL-10 in LPS-stimulated macrophages, which suggests that this compound could be an alternative in the treatment of degenerative diseases associated with inflammation, such as rheumatoid arthritis and other chronic diseases.

The lysosome is a cell organelle that produces some enzymes which are involved in inflammatory process, and the membrane of erythrocytes is similar to lysosomal membrane. Erythrocytes in hypotonic solution are lysed, resulting in the leakage of serum protein and fluids into the tissue, and in turn activating an acute inflammatory process and tissue injury [29], which can be prevented by the stabilization of the membrane; therefore, compounds that inhibit the lysis of red blood cells are candidates as anti-inflammatory agents [30,31,32]. DHAP diminished the lysis of erythrocyte membranes induced by hypotonic solution, and therefore this compound might reduce the production of phospholipase through the inhibition of various inflammatory mediators [31].

In several species of the *Artemisia* genus have been found p-hydroxyacetophenone. Recently Ching-Wen et al. demonstrated that this compound has anti-inflammatory and antinociceptive activities [33]. Other authors mentioned that due to antioxidant and anti-inflammatory effects of compounds, chemicals related with p-hydroxy acetophenone could be use as hepatoprotective compounds [34]. Later Neng-Hua et al. [35] isolated 10 acetophenones from *Acronychia oligoplebia* and studied their anti-inflammatory and antioxidant activities. One of these compounds was 3,5-diprenyl-1-4-dihydroxy-6-methoxyacetophenone showed anti-inflammatory activity, and is structurally similar to DHAP. This compound was additionally tested using DPPH radical-scavenging capacity showing antioxidant activity with IC_50_ value of 0.15 mM, which is similar to that obtained for DHAP (IC_50_ = 0.096 mM).

The results obtained in this study indicate that DHAP might be used in the treatment of inflammatory problems.

## 4. Materials and Methods

### 4.1. Chemicals

12-O-tetradecanoylphorbol-13-acetate (TPA), IND, diclofenac, Dulbecco’s Modified Eagle’s Medium (DMEM), fetal bovine serum (FBS), antibiotics, 3-(4,5-dimethylthiazol-2-yl)-2,5-diphenyl tetrazolium bromide (MTT), Griess reagent, dimethyl sulfoxide (DMSO), lipopolysaccharide (LPS), Tetramethyl silane (TMS) and Silica gel 60, and 1,1-diphenyl-2-picrylhydrazyl radical (DPPH) were purchased from Sigma Aldrich. Dichloromethane (DCM), ethyl acetate (EtOAc), acetone, and hexane were purchased from Chemical Lufra. Murine J774A.1 macrophages were purchased from ATCC. Immunoenzymatic kits for the quantification of interleukin (IL) 1β were purchased from PeproTech, and kits for the quantification of IL-6, IL-10, and tumor necrosis factor alpha (TNF-α) from Tonbo Biosciences.

### 4.2. Plant Material

The aerial parts (steam and leaves) of *A. pazcuarensis* were collected in the municipality of Huitzilac, state of Morelos, in August 2020. The plant was identified by M. en C. Gabriel Flores Franco, and a specimen was placed in the herbarium of the Autonomous University of the State of Morelos (UAEM) (voucher number HUMO30367).

### 4.3. Extraction and Isolation

The aerial parts of *A. pazcuarensis* were dried at room temperature. After grinding, 500 g of crushed vegetal material was extracted by maceration with 5 L hexane for 3 days to remove waxes and fats. The mixture was filtered, and the solvent was evaporated on a rotary evaporator under reduced pressure. Subsequently, the plant material was extracted by maceration with 5 L of DCM for 3 days. The mixture was filtered, and DCM was eliminated. The organic extract recovered with DCM was separated by open-column chromatography packed with silica gel (Macherey-Nagel 60, 70-230 mesh ASTM) using hexane as the mobile phase, and the polarity was increased little by little adding small amounts of EtOAc. A white crystalline solid was obtained, and the purity of the crystals was determined by thin layer chromatography. The structure of the pure compound was determined by IR, NMR, and GC-MS.

### 4.4. Structural Analysis

NMR mono- and bi-dimensional spectra were recorded at 25 °C in a spectrometer Agilent DD2, 600 MHz with the OneNMR probe. The sample was dissolved in CDCl_3_ and the chemical shifts of ^1^H and ^13^C were reported using TMS as reference. Infrared spectra were obtained in a Spectrum Two FT-IR Spectrometer (Perkin Elmer) with ATR, and the samples were analyzed by forming a film with chloroform. The GC-MS analysis was record in an Agilent Technologies, and the spectral data were digitalized using the Mass Spectrum Digitizer program from the National Institute of Standards and Technology (NIST). The UV spectrum was obtained in a Perkin Elmer Lambda 35 spectrophotometer (MeOH), scanning was performed from 200 to 400 nm.

### 4.5. Animals

Male CD-1 mice (25–30 g) were provided by the Universidad Autónoma Metropolitana (UAM) animal facility. The animals were housed at 20–22 °C, under 12:12 h light-dark cycles, and water and food were supplied ad libitum. The experimental protocol number 140 was approved by the Research Bioethics Committee of the UAM. All experiments were carried out in accordance with the current procedure for the care of experimental animals established by the Official Mexican Standard (NOM-062-ZOO-1999).

### 4.6. Acute Anti-Inflammatory Activity: Edema Induced by TPA

Groups of eight animals were used. The right ear of each mouse received a topical application of 2.5 µg/mouse of TPA dissolved in acetone (20 µL). Thirty minutes after the TPA application, was topical administered at a dose of 2 mg/ear of IND (reference drug group), or 2 mg/ear of DHAP (test group), or 20 µL of acetone as vehicle (negative control group) to the right ear of the mice. The animals were euthanized 6 h after TPA administration, and central segments of 6 mm were obtained from the two ears of each animal and weighed to calculate the percentage inhibition of edema [36].

### 4.7. Antioxidant Activity by DPPH Assay

The free radical scavenging activity of DHAP was determined by DPPH assay [37]. 100 µL of a methanol solution of DPPH (0.208 mM) were mixed with 100 µL of DHAP or ascorbic acid (positive control) dissolved in methanol (6.25, 12.5, 25, 50 and 100 µg/mL). The reaction mixture was incubated in dark for 20 min, after this time the absorbance was determined at 490 nm. Each determination was performed triplicate. The percentage of inhibition was calculated by following equation.

Inhibition (%)=[1−(As/Ac)]×100

where As = Absorbance of sample (DHAP); Ac = Absorbance of positive control (ascorbic acid).

The inhibition effect of DHAP on DPPH radical scavenging activity was obtained by lineal regression using the graph of percentage of inhibition of DPPH versus DHAP and expressed as IC_50_ in mg/mL.

### 4.8. Cell Viability Assay

J774A.1 macrophages were seeded per well in 96-well plates in DMEM at a density of 5 × 10^3^ cells per well. After 24 h of incubation, the cells were treated with a concentration of 1 to 200 µg/mL (3.67, 18.37, 36.74, 91.85, 183.70, 368.75, and 734.24 µM) DHAP. This solution was prepared from a mixture of 5 mg of DHAP dissolved in 1 mL of PBS (phosphate buffer). Untreated cells were used as a negative control. The compound was dissolved in PBS. After 48 h of treatment, 10 µL of MTT at a concentration of 5 mg/mL were added. Plates were then incubated for 4 h at 37 °C. Subsequently, the medium was removed, and the formazan crystals were dissolved in DMSO. Optical density was determined at 540 nm. Six replicates were used for each group to determine viability. The IC_50_ was calculated by linear regression analysis [38].

### 4.9. Determination of Nitric Oxide (NO) and Cytokines

J774A.1 macrophages were seeded in 6-well plates (5 × 10^5^ cells per well), stimulated with LPS (5 μg/mL), and incubated for 2 h. Subsequently, 91.78 μM of DHAP, IND (47.79 μM), or vehicle were added. The supernatants were collected after 24 h and stored at −70 °C until further analysis. The concentrations of IL-1β, IL-6, IL-10, and TNF-α in the supernatants were determined using a commercial ELISA kit according to the manufacturer’s instructions. The absorbance was recorded with a microplate reader at 405 nm. For the determination of NO, 100 µL of the supernatant were mixed with 100 µL of Griess reagent, the mixture was incubated at 37 °C for 30 min, and the absorbance was recorded at 540 nm. The % reduction in NO production was determined in relation to the amount produced by the group treated only with LPS [39].

### 4.10. Membrane Stabilization Property

Blood samples were obtained from healthy human volunteers who had not ingested steroids or NSAIDs or contraceptive drugs for two weeks. The samples were washed with Alsever’s solution (2% dextrose, 0.8% sodium citrate, 0.05% citric acid, and 0.42% sodium chloride in water) after which the mixture was centrifuged for 10 min at 3000 rpm. Subsequently the cells were washed with Alsever’s solution three times. Five different concentrations of DHAP or diclofenac (25–200 µg/mL) were prepared in PBS [31,32]. PBS buffers were used as the negative control. Then, the mixtures were incubated for 30 min at 37 °C, after which the samples were centrifuged at 3500 rpm for 5 min and the supernatants were read at 540 nm [11]. The percentage of protection was calculated using the following formula.

$$\%\;\mathrm{Protection}=100-\left(\frac{\mathrm{Optical}\;\mathrm{density}\;\mathrm{of}\;\mathrm{Test}\;\mathrm{sample}}{\mathrm{Optical}\;\mathrm{density}\;\mathrm{of}\;\mathrm{Control}}\times 100\right)$$

### 4.11. Statistical Analysis

The results are expressed as the mean ± SEM. Statistical analyses were performed by ANOVA and Tukey’s post hoc test, using the statistical program iner-STAT20-a v1.3. Values of * *p* < 0.05 and ** *p* < 0.01 were considered statistically significant.

## 5. Conclusions

The results of this study demonstrate that DHAP possesses in vitro and in vivo anti-inflammatory activity by reduction of edema earing mouse induced by TPA and inflammatory mediators such as NO, IL-1β, IL-6 and TNF-α, and promoting the release of the anti-inflammatory cytokine IL-10 and protected membrane lysis. In further studies, we will continue to research of the mechanisms of action of this compound.

## Figures and Tables

**Table 1:** Anti-inflammatory activity of DHAP on ear edema induced by TPA in mice.

Treatment	Dose (mg/ear)	Difference of Weight (mg)	% Decrease in Inflammation
Negative group	---	11.85 ± 0.57 **	0.0
DHAP	2.0	3.54 ± 0.66 *	70.10 ± 5.53
	1.0	4.47 ± 0.29 *^,^**	62.27 ± 2.48
	0.5	5.74 ± 0.36 *^,^**	51.54 ± 3.00
Indomethacin	2.0	2.76 ± 0.17 *	76.73 ± 1.41

The values are the mean ± S.E.M. *(n* = 8). * *p* < 0.001 statistically significant difference compared with negative group and ** *p* < 0.001 statistically significant difference compared with IND group.

**Table 2:** Percent human red blood cell membrane stabilization by DHAP.

μg/mL	Diclofenac	DHAP
400	87.82 ± 0.42	75.70 ± 2.79
200	77.85 ± 0.31	76.17 ± 0.51
100	84.97 ± 0.45	83.69 ± 0.83
50	84.19 ± 0.10	85.35 ± 0.74
25	87.93 ± 0.56	86.59 ± 0.96

The values are the mean ± S.E.M. (n = 4). The absorbances are shown in Supplemental Material (Table S1).

## Data Availability

Data is contained within article and the Supplementary Materials.

## Supplementary Materials

The following supporting information can be downloaded at: https://www.mdpi.com/article/10.3390/ijms232315012/s1.
