# Supplementary material for: Anti-Inflammatory Activity of 3, 5-Diprenyl-4-hydroxyacetophenone Isolated from Ageratina pazcuarensis
Source: Int J Mol Sci. 2022 Nov 30;23(23):15012. doi: 10.3390/ijms232315012 (PMC9741312; doi:10.3390/ijms232315012)
Supplement: Supplementary file 1 [file ijms-23-15012-s001.zip › ijms-1961841-supplementary.pdf]

Anti-inflammatory activity of 3, 5-diprenyl-4-hydroxyacetophenone isolated from *Ageratina pazcuarensis*.

Sarai Rojas-Jiménez, María Salud Pérez-Gutiérrez, Ernesto Sánchez-Mendoza, Rubria Marlene Martínez-Casares, Nimsi Campos Xolalpa, María Guadalupe Valladares-Cisneros, David Osvaldo Salinas-Sánchez.

Spectroscopy and Spectrometry data of 3, 5-diprenyl-4-hydroxyacetophenone.

- **NMR Spectra**

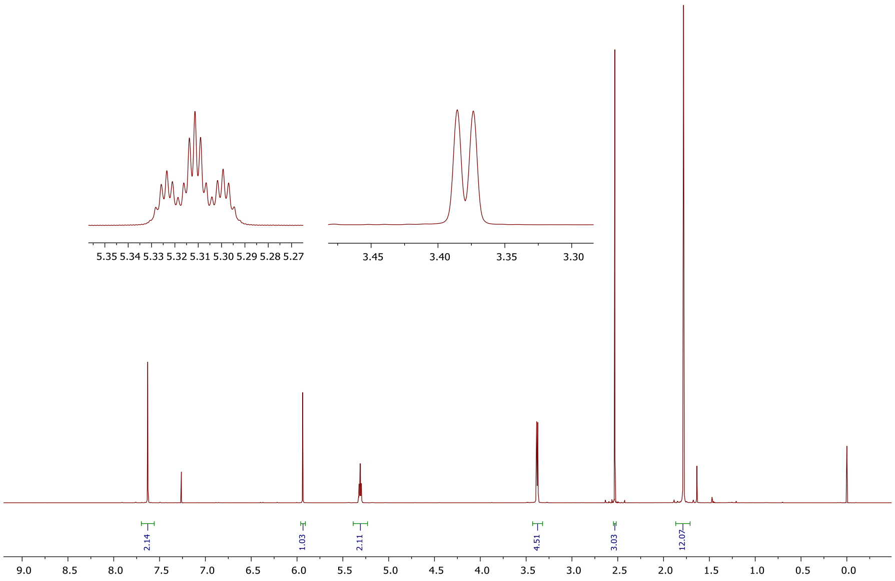

Figure S1, NMR-^{1}H spectrum.

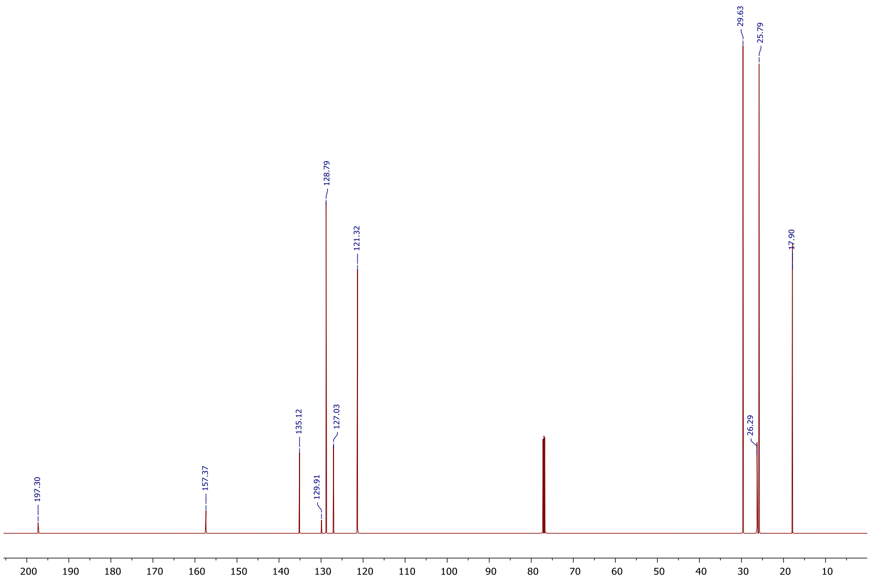

Figure S2, NMR-^{13}C spectrum.

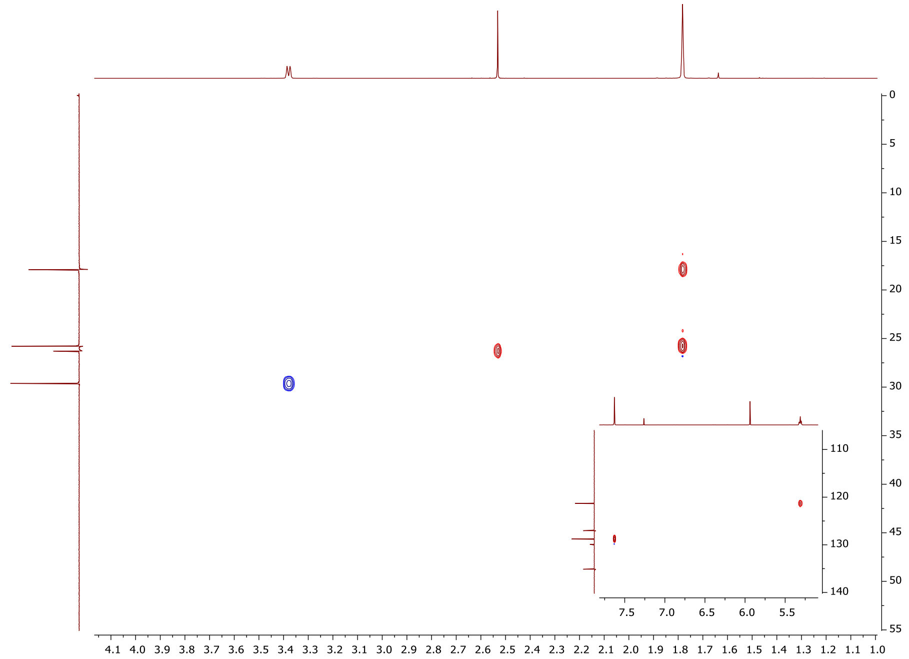

Figure S3, gHSQC NMR spectrum.

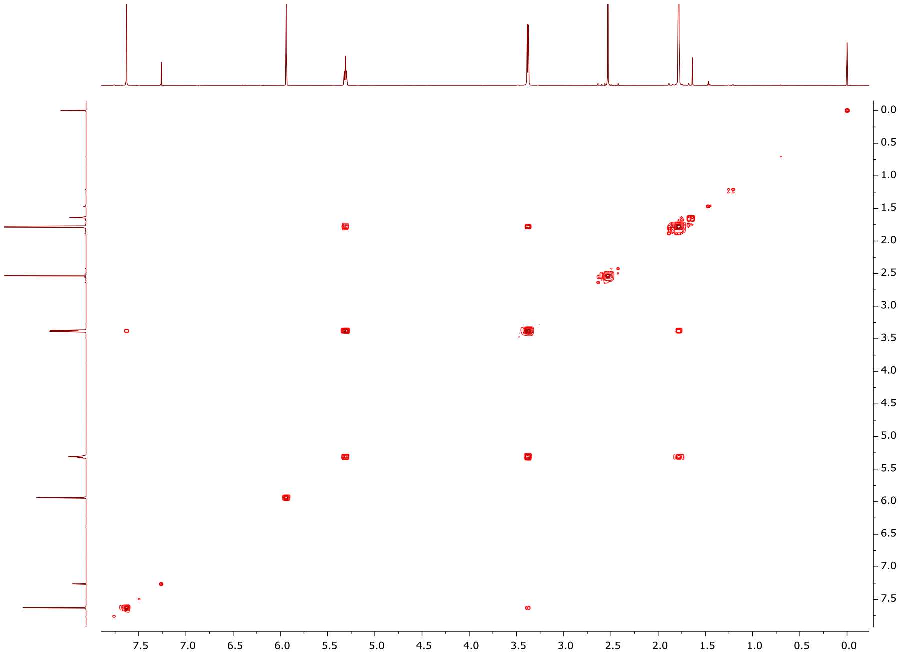

Figure S4, COSY NMR spectrum.

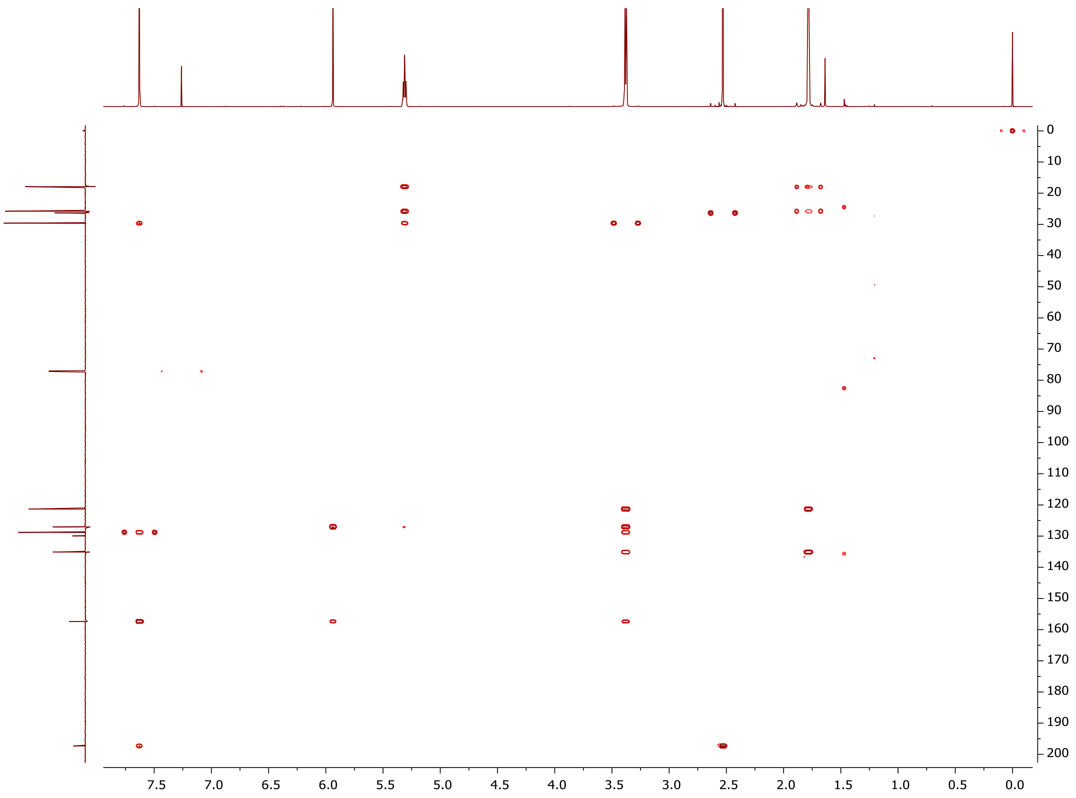

Figure S5, gHMBC NMR spectrum.

- **IR-Data**

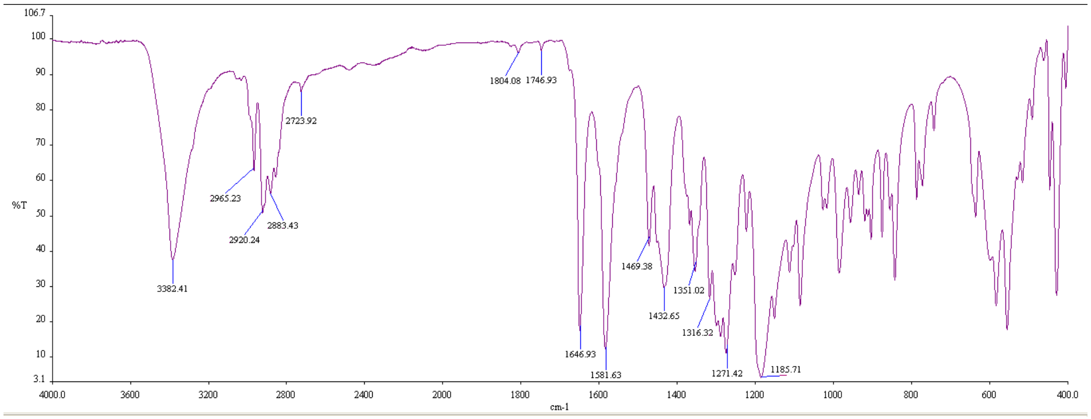

Figure S6. FT-IR Spectrum

- **Gas Chromatography and Mass Spectrometry Data**

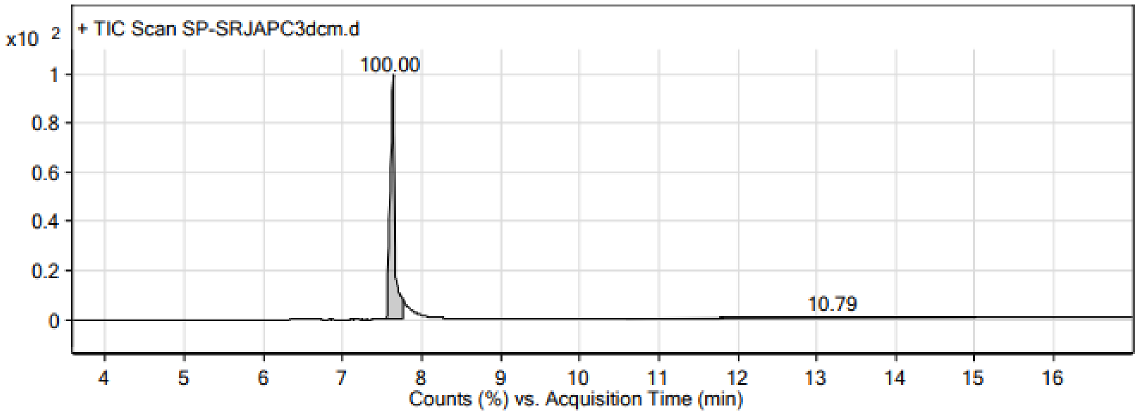

Figure S7. Gas chromatogram. T_R=7.8 min.

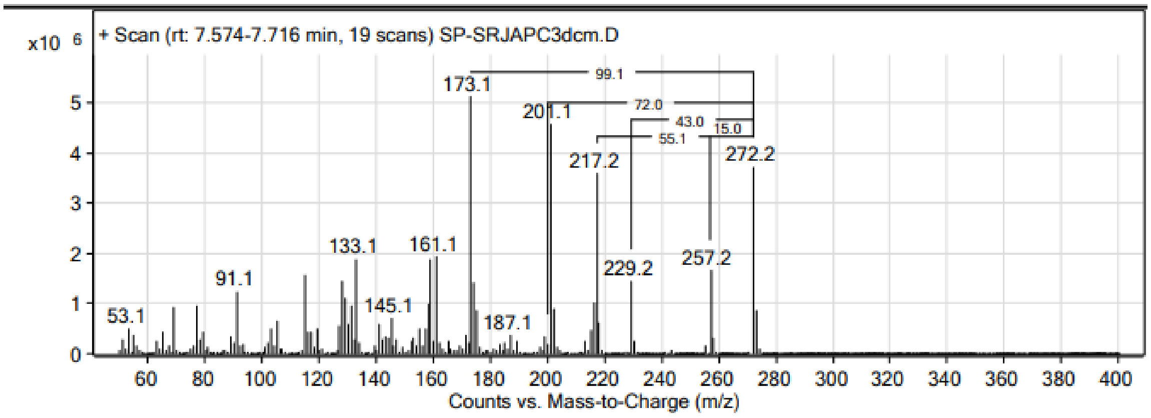

Figure S8. EI-MS Spectrum (70 eV).

Table S1. Absorbances obtained in cell membrane stabilization assay.

Concentration (µg/mL)	Absorbance	
	Diclofenac	DHAP
400	0.081 ± 0.003	0.162 ± 0.015
200	0.148 ± 0.002	0.159 ± 0.004
100	0.100 ± 0.003	0.109 ± 0.006
50	0.106 ± 0.001	0.098 ± 0.005
25	0.081 ± 0.004	0.090 ± 0.007
